# A New GLLD Operator for Mass Detection in Digital Mammograms

**DOI:** 10.1155/2012/765649

**Published:** 2012-12-22

**Authors:** N. Gargouri, A. Dammak Masmoudi, D. Sellami Masmoudi, R. Abid

**Affiliations:** ^1^Computer Imaging and Electronic System Group, CEM Laboratory, Department of Electrical Engineering, Sfax Engineering School, University of Sfax, P.O. Box 1169, 3038 Sfax, Tunisia; ^2^El Farabi Radiology Center, 14 Janvier Avenue, 3000 Sfax, Tunisia

## Abstract

During the last decade, several works have dealt with computer automatic diagnosis (CAD) of masses in digital mammograms. Generally, the main difficulty remains the detection of masses. This work proposes an efficient methodology for mass detection based on a new local feature extraction. Local binary pattern (LBP) operator and its variants proposed by Ojala are a powerful tool for textures classification. However, it has been proved that such operators are not able to model at their own texture masses. We propose in this paper a new local pattern model named gray level and local difference (GLLD) where we take into consideration absolute gray level values as well as local difference as local binary features. Artificial neural networks (ANNs), support vector machine (SVM), and k-nearest neighbors (kNNs) are, then, used for classifying masses from nonmasses, illustrating better performance of ANN classifier. We have used 1000 regions of interest (ROIs) obtained from the Digital Database for Screening Mammography (DDSM). The area under the curve of the corresponding approach has been found to be *A*_*z*_ = 0.95 for the mass detection step. A comparative study with previous approaches proves that our approach offers the best performances.

## 1. Introduction

 Breast cancer is the major public health problem in the world. It constitutes the most common cancer among the female population [1]. A study developed by American cancer society estimates that between one in eight and one in twelve will be diagnosed with breast cancer in their life time [2]. The European community estimates that breast cancer corresponds to 19% of cancer death. Moreover, it represents 24% of cancer cases [3]. In a Tunisian country, breast cancer is 16,5% of cancer death [4]. Mostly, 25% of all cases of breast cancer deaths occur if women were diagnosed between the age of 40 and 49. Although breast cancer incidence has increased over the last decade, breast cancer mortality has declined among women of all ages [5], thanks to the development of both breast cancer treatment and mammography screening.

Among the different imaging modalities used for the detection of breast cancer, mammography remains the most used one to reveal breast abnormalities. Vacek et al. [14] demonstrate that the ratio of breast tumor detection in Vermont (USA), when applying screening mammography, increased from 2% to 36% between 1995 and 1999.

Nowadays, the digital mammography gives the opportunity of increasing the use of the CAD systems in order to help the expert radiologists in the interpretation and diagnosis of mammograms [15].

However, the rapid improvement of full digital mammography has been accompanied by natural increase of such systems. The CAD is a set of tools developed to help radiologists in the detection and interpretation of mammographic images [16].

Back in 2001, Freer and Ulissey [16] have proposed an algorithm using substantial dataset containing 12,860 cases and have concluded that the application of CAD in the analysis of screening mammograms may increase the malignancies detection at an early stage. The main disadvantage of existent CAD systems is the lack of general algorithms producing good results for all cases and images. Masses and microcalcifications are common lesions found in mammographic images. We will focus in this paper on mass-related lesions. In order to develop an improved computer-aided clinical decision classifying the tumor and identifying the stage of the cancer, we must ensure whether it is an area which contains a mass or not. The mass detection is therefore a valuable step in diagnosis. Our work focuses on classification of the tissue of the breast as mass or mass free. So, mass detection system is able to assist health professionals in finding out mass abnormalities in mammograms.

Several algorithms are typically based on only one view. However, some recent approaches have used multiple views [17], but this has three main drawbacks.

- Image views must be properly registered to allow a correct comparison of regions.
- Gray level values must also be correctly registered.
- There are some cases where comparison is not possible, because no correspondance between pixels can be done (e.g., the case of patients which have suffered from a previous breast surgery).

It is important to note that algorithms typically working with one view can always be applied to multiple views. Textural information has already been used to solve this problem and has been introduced in several works [6, 10]. Oliver et al. have proposed an algorithm for mammographic mass detection based on LBP [18]. Results have indicated that the use of LBP and its extensions has been promising in different comparative studies and has been applied in different texture analysis tasks [6, 19]. However, LBP descriptors are not able to model mammogram texture because they are mapping only the differences of pixel gray level values. So, we will work here on a new approach taking into consideration the texture whole information, the local difference and local gray values as features, namely, gray level and local difference features (GLLD). Accordingly, we intend to investigate the efficiency of the GLLD based approach as a method of feature extraction. We perform our experiments on a set of 1000 ROIs obtained from the DDSM database.

This paper, using a single view, proposes a new CAD methodology in order to achieve better performances in terms of false negative and false positive using GLLD operator. The remaining of this paper is organized as follows.  Section 2 shows some related works on mass detection in mammogram images. In Section 3, we present a brief review of LBP operator and an analysis of our GLLD based approach. In Section 4, a brief description of a set of classification methods is given, namely, support vector machine (SVM), k-nearest neighbors (kNNs), and artificial neural network.  Section 5 is reserved to the validation of the GLLD proposed technique simulation, and results and discussion are conducted. In the last section, we summarize the paper contribution and end our work by some concluding remarks and future work.

## 2. Background

Several image processing techniques have been formulated as tools that can assist early automatic mass detection [20, 21]. Algorithms for mammographic mass detection using a single image view are based on a characteristic classifier scheme: for a given database consisting of known cases, the decision making system learns how to distinguish between the two kinds of ROIs (mass and nonmass ROI). Thereafter, once the given system has been trained, a new ROI can be rightly classified. Among all these detection algorithms, we can differentiate between two strategies. The first one includes the algorithms which extract features usually related to their texture from the ROI and then trains a classifier. Approches lying in such strategy are summarized in Table 1. The second strategy turns this problem into a template matching one. Each new ROI is compared to all the remaining ROI images obtained from the database in order to be finally classified as mass or nonmass.  Table 2 shows different approaches applying this strategy.

On the one hand, Qian et al. [6] have analyzed the implementation of an adaptive CAD to develop a fully automatic procedure for mass segmentation and classification which consists in training a novel Kalman-filtering neural network to classify features extracted from wavelet decomposition [6]. On the other hand, Christoyianni et al. [7] have extracted features based on independent component analysis (ICA), gray level and texture, in order to train the ANN as a classifier. Furthermore, they have applied the principal component analysis (PCA) for the preprocessing step to overcome the problem of complexity and of increasing dimensionality. Oliver et al. [8] have proposed a different strategy. The latter is based on the translation of eigenfaces approach for face detection/classification problems to the mass detection one. They introduced the concept of spanning the ROI subspace of an original image space. As result of such transformation, they have obtained a vector which describes the contribution of each eigenrau for the representation of the corresponding image. They have used these vectors in the construction of the models for the step of training. In [9], Oliver et al. have extended their proposed method based on PCA by using the two-dimensional PCA (2DPCA) technique. Varela et al. [10] have proposed a methodology based on extracting gray level as well as morphological features and classifying, using ANN, the new ROI. Leonardo et al. [11] have proposed an algorithm for the detection of masses in mammographic images. The technique is based on the use of textural and shape measures for K-means clustering algorithm and the SVM, aiming at detecting masses in mammographic images. 

As shown in Table 2, the proposed approach of Chang et al. [12] and Tourassi et al. [13] has been based on a template matching-based approach. They have proposed for classification purposes to undertake a comparison of the new ROI with the remaining ROIs in the database composed of ROIs depicting masses. The difference between these works appears in the similarity measure function. As indicated in Table 2, there are only limited publications trying to detect masses using template-matching based methods. From the two tables, we can conclude that one of the main dissimilarity among these recent works is the ratio between the ROIs depicting abnormality and the total number of cropped images. It is important to note that when the number of normal ROIs increases, the number of ROIs wrongly classified is likely to increase. One should remember that the purpose of this work is the classification of mammographic masses and normal breast tissue. All the developed methods allow the tradeoff between the reduction of false positive fraction and the increase of false negative fraction. Such trade off can be ensured when using the receiver operating characteristics (ROC) [22] in the performance evaluation step. This is the case of most of the approaches in Tables 1 and 2. The ROC curve is a graphical curve representing the true positive rate (sensitivity) versus the false positive rate (100 specificity), extensively used in classifier performance evaluation. Points representing ROC curve correspond to sensitivity/specificity pair representing a particular decision threshold. The AUC (known as *A*_*z*_) is an information about the overall performance of the approach. Furthermore, the latter is a metric which can be used to compare different features, and it allows the reduction of the ROC curve to a single value summarizing expected performance. A reasonable test should have

(1) 0.5°≤AUC°≤1.

Most of the approaches in the first strategy have the drawback that a large number of features need to be calculated but only the most discriminant will be selected [7, 8]. Besides, for the second strategy, the used similarity function measure for classifying needs to be recomputed for each element. In our paper, to overcome such limitations, the LBP operator has been investigated with the idea of performing gray scale invariant texture analysis. The latter has proved to be relevant in many applications. However, it shows some limitations when applied to mammographic image. For instance, it gives the same results with two different absolute gray levels. Knowing that the gray level information is of great importance in mammography, our approach will add to LBP absolute gray level information rather than gray level difference. We will focus in our approach on making use of small size feature vector as well as possible. In the following section, we will introduce our mass detection methodology based on the GLLD for the extraction of texture features obtained from the ROIs. 

## 3. Local LBP Approach and Improvements

 Texture classification is nowadays a challenging problem. It is an active topic in computer vision research. Early methods of texture classification are based on statistical analysis of images with different textures. The most representative ones are the cooccurrence matrix method [23] and filtering for texture-classification methods [24]. At an early stage, exploratory models were developed to investigate rotation invariance in texture classification, such as hidden Markov model [25] and Gaussian Markov random field [26]. Varma and Zisserman [27] have proposed to learn from a training set a rotation invariant texton and to classify the obtained texture according to its texton distribution. Varma and Zisserman [28] have later proposed to use the image patch in order to represent features directly. Some recently proposed works have been developed for scale as well as affine invariant texture classification. Later, Ojala et al. [29] have proposed the LBP histogram application in order to achieve a rotation invariant texture classification. It is worth noting that the LBP is efficient in describing local image pattern and its performance in computer vision and pattern recognition is promising. However, it still needs to be improved for mammography texture modeling. In order to generate texton, Ojala et al. [30] have applied the Absolute Gray Level Difference (AGLD) between each pixel and its neighbors. After that, the obtained histogram has been used to represent the image texture. Then, Ojala et al. [29] have proposed the LBP using the sign of the difference for the representation of local patterns. In [31], Ojala et al. suggested to use signed gray level Difference (SGLD) and its multidimensional distribution for the description of texture and considered LBP as a simplification of SGLD. With such variants of LBP, there still remain questions that need answering, such as what information is lost in the considered code? How to represent the missing information to obtain better texture modeling? Here, we propose a new feature extractor to improve the system performance, based on GLLD features.

### 3.1. Brief Review of LBP Formulation

 The LBP operator used eight neighboring pixels when considering the center gray value as threshold. This operator generates “1” if the considered neighbor value is greater or equal to that of the center. Otherwise, it generates “0.”

**Figure 1:**
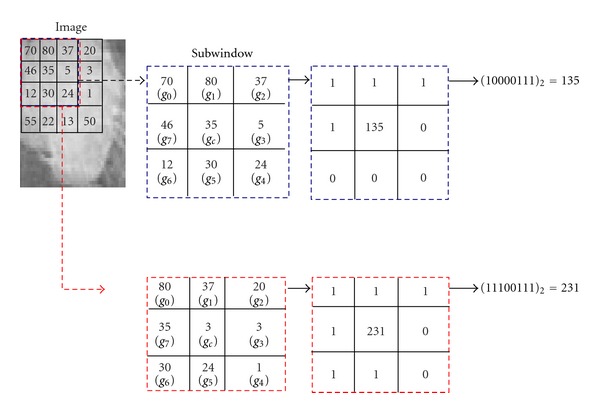
Example of basic LBP operator.

Accordingly and referring to Figure 1, LBP [29] code may be computed as follows:

(2) LBPP,R=∑p=0P−1s(gp−gc)2p,s(x)={1,x≥00,x<0,

where *g*_*c*_ corresponds to the gray value of the central pixel, *g*_*p*_ corresponds to the value of its neighbors, and (*p* = 0,1,…, *P* − 1) and *P*, *R* correspond to the number of neighbors and to the radius of the neighborhood, respectively. The binary code is then represented with an 8-bit number. *g*_*p*_ coordinates are ((*R* cos⁡(2*πp*/*P*) and *R* sin(2*πp*/*P*)). If neighbors are not in the image grids, their gray values may be estimated by interpolation. After identifying LBP pattern of each pixel (*i*, *j*), we associate LBP histogram to the whole image, with a given image size (*N*1∗*N*2) as

(3) HLBP(k)=∑i=1 N1∑j=1N2f(LBPP,R(i,j),k), k∈[0,K],f(x,y)={1,x=y0,otherwise,

where *K* corresponds to the maximum gray level value.

Let *U* be a function corresponding to the value of an LBP pattern, it is defined as the number of transitions (i.e., change from 0 to 1 or 1 to 0) in the following pattern:

(4) U(LBPP,R)=|s(gp−1−gc)−s(g0−gc)| +∑p=1P−1|s(gp−gc)−s(gp−1−gc)|.

Patterns corresponding to limited transitions or discontinuities are with *U* ≤ 2, in a binary presentation. Otherwise, these patterns are noted as uniform LBP patterns [29]. The mapping from the original LBP_*P*,*R*_ to LBP_*P*,*R*_^*u*2^, knowing that the superscript *u*2 refers to uniform patterns, may be implemented using a look-up table containing 2^*p*^ elements.

A local rotation invariant pattern is defined as follows [32]:

(5) LBPP,Rriu2={∑p=0P−1|s(gp−gc)|if  U(LBPP,R)≤2P+1otherwise.

The mapping from LBP_*P*,*R*_ to LBP_*P*,*R*_^riu2^, knowing that the superscript riu2 corresponds to rotation invariant uniform patterns, may be implemented using a look up table.

### 3.2. GLLD Feature-Based Approach

The main limitation using LBP code is that it may give the same results with two completely different gray levels when the differences with the neighbors are the same.

Knowing that for mammographic images, the gray level information is directly related to the breast tissue density, gray level and local difference are two important features of the texture which must be used together in order to have more accurate results.

In our approach, we propose to calculate the average for each 3 × 3 neighborhood and to attribute it to the central pixel. The new value of the central pixel is noted as *g*_*c* mean_. 

**Figure 2:**
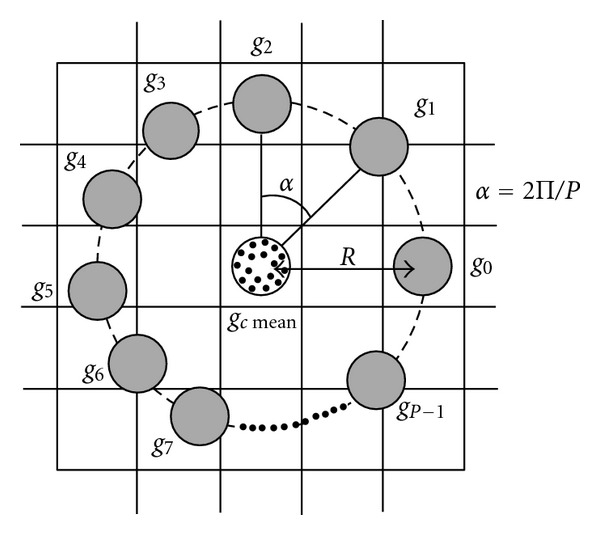
The central pixel *g*_*c*_ and its *P* circularly symmetric neighbor with radius *R*.

 Given the new value of the central pixel *g*_*c* mean_ and its *P* circularly symmetric neighbor (see Figure 2), the substraction of the value of *g*_*c* mean_ is presented as follows:

(6) T=t(gc mean,g0−gc mean,…,gP−1−gc mean).

Thus, the difference between *g*_*c* mean_ and *g*_*p*_ may be represented as diff_*p*_ = *g*_*p*_ − *g*_*c* mean_ and the local difference may be represented with a vector noted diff_*p*_ knowing that diff_*p*_ = [diff_0_,…, diff_*P*−1_], diff_*p*_ describes the local image structure around the *g*_*c* mean_. Because of its robustness and efficiency, the obtained vector diff_*p*_ is decomposed of sign and modulus components in order to achieve much better performance in texture classification. In our proposal, *s*_*p*_ corresponds to the sign of the differences, and it is obtained by thresholding with respect to the value of *g*_*c* mean_ as expressed in (8). However, *m*_*p*_ corresponds to the absolute value of diff_*p*_ as expressed in (9). We obtain, also, two vectors, the sign vector [*s*_0_,…, *s*_*P*−1_] and the modulus vector [*m*_0_,…, *m*_*P*−1_], with

(7) diffp=mp·sp,

knowing that

(8) sp={1,diffp≥0−1,diffp<0,

(9) $$\begin{array}{c} m_{p}=\left|\text{dif}{\text{f}}_{p}\right|. \end{array}$$

**Figure 3:**
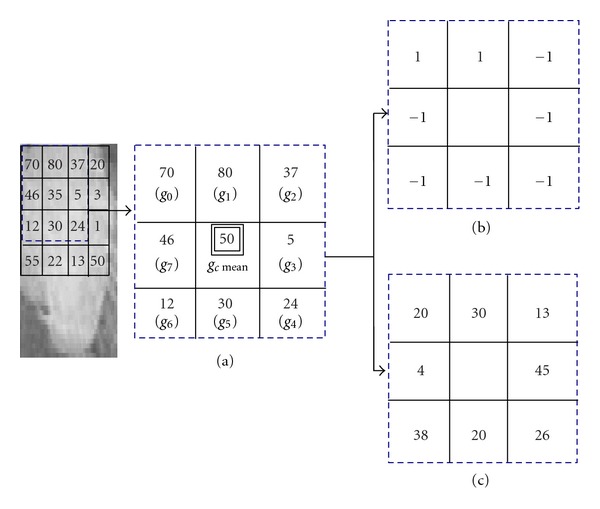
(a) A 3∗3 block with central pixel corresponding to the mean value of its neighbors. (b) The sign components. (c) The magnitude components.

Figure 3 shows an illustration example of the proposed method. Aiming at recognizing robustly and efficiently the texture patterns, we should extract both absolute and relative features from pixel gray levels.

The modulus component provides discriminant information to the sign component; the intensity value of the central pixel corresponding to the mean value of its neighbors may also give us useful information [33, 34]. It will also be seen that by coding the sign, the modulus, and the central gray level features into rotation invariant binary codes and fusing them, results may provide much better performance in mammogram texture classification than using each one by itself. This fusion provides useful information about local gray level which is so important in the stage of mass detection in mammographic images. 

#### 3.2.1. SGLLD, MGLLD, and CGLLD Operators

**Figure 4:**
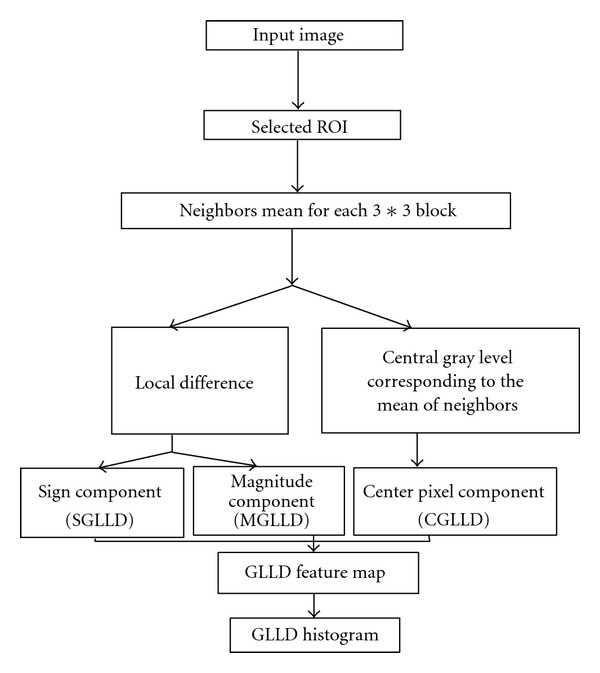
Different processing steps of of the proposed GLLD based approach.

 In this subsection, we present the gray level and local difference (GLLD) different processing steps to explore the proposed three features, which are illustrated in Figure 4. 

 We start by extracting different ROIs from mammographic images. After that, in the selected ROI, each central gray level corresponds to the mean of its neighbors and its local difference. The latter is decomposed into sign and modulus components as expressed in (7). Given a pixel in the image, the sign coding component is noted as (SGLLD) and is computed by comparing it with the values of its neighbors as follows:

(10) SGLLDP,R=∑p=0P−1s(gp−gc mean)2p,

where *s*(*x*) is defined by

(11) s(x)={1,x≥0−1,x<0,

where *g*_*c* mean_ is the average value of the central pixel and its neighbors. Inspired by the method of coding (SGLLD), the coding of the Magnitude component is noted as (MGLLD) and is defined as follows:

(12) MGLLDP,R=∑p=0P−1t(mp,c)2p,

(13) t(x,c)={1,x≥c0,x<c,

where *c* corresponds to a global gray level threshold which is determined adaptively. We set it as the average value from the whole image.

The new value of the central pixel, which expresses the gray level of the image, represents also a discriminant information. So, to make it consistent with the two previous operators SGLLD and MGLLD, we code it as

**Figure 5:**
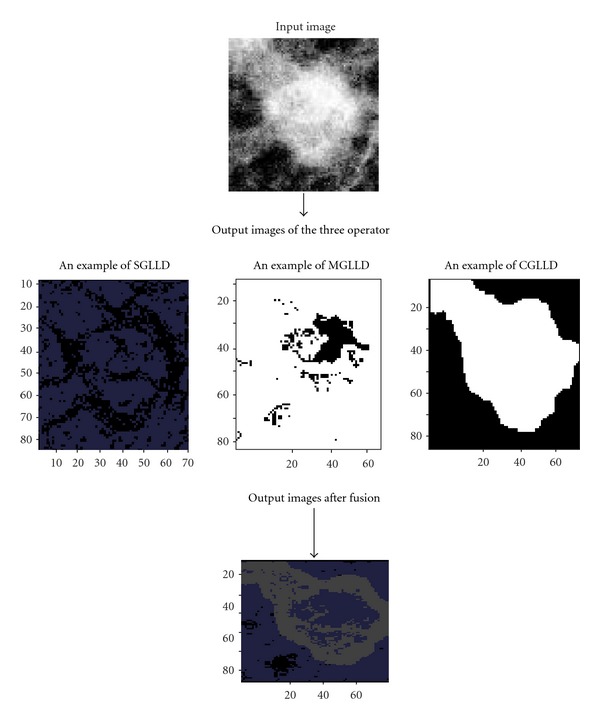
The image results after the application of the three operator and their fusion.

(14) CGLLDP,R=t(gc mean,cI),

where *t* is already defined in (13), *c*_*I*_ corresponds to the threshold and is set as the mean gray level of the whole input image. CGLLD is defined to extract the image local gray level.  Figure 5 illustrates the image results after the application of the three operators and their fusion. 

**Figure 6:**
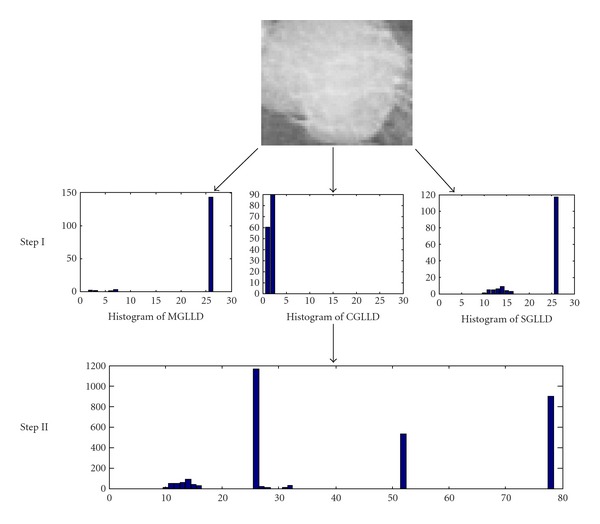
The extraction of the different local features from an ROI sample. Step (I): the texture features can be computed by building the histogram over the corresponding ROI. Step (II): the histogram from the three operators is concatenated to build the texture features of the selected ROI.

 For the three obtained codes, the rotation invariant version is defined to achieve rotation invariant classification. Each code carries specific texture information, that is why we concatenate them to build the GLLD feature, which corresponds to a vector. So, the three obtained histograms were concatenated to one histogram (cf.  Figure 6). 

 The procedure consists in using the GLLD in order to build local descriptor of the obtained ROIs knowing that the concatenation leads to global description and the obtained global and local GLLD texture descriptor are, then, used as features for mass detection.

**Figure 7:**
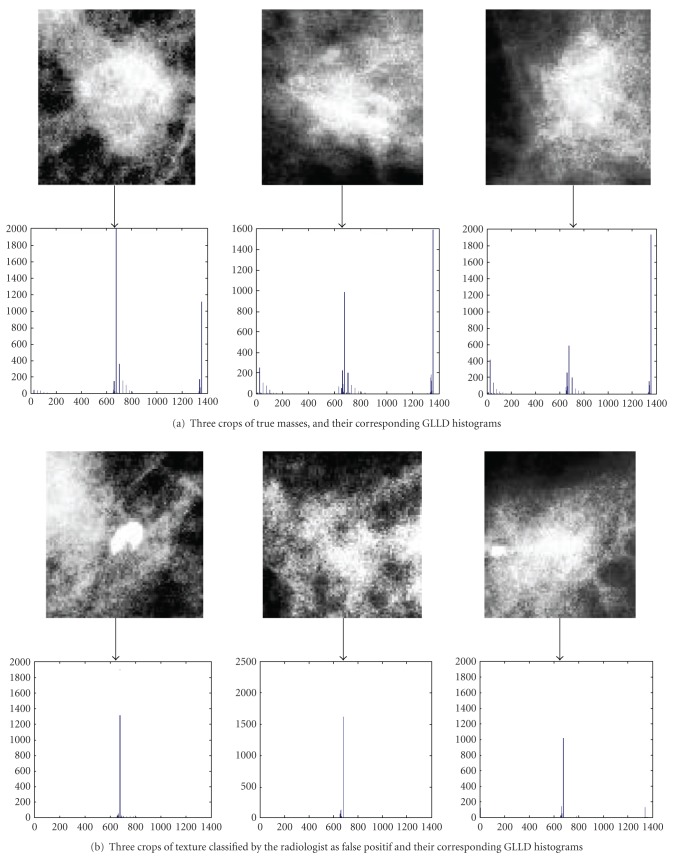
GLLD feature distributions extracted and concatenated to constitute the final histogram.

The following images (Figure 7) illustrate the obtained histogram for differents ROIs. In this figure, we have considered three trivial examples as well as three challenging examples which were misclassified by a radiologist. All of the six ROI examples where correctly classified by our GLLD texture features. In a further section, we will focus on validating our approach statistically on the DDSM database.

## 4. Classification

 The last step of our proposal is mass classification. For the sake of generality and for doing a best choice of the classifier, an investigation of three classifiers will be undertaken, namely, support vector machine (SVM), k-nearest neighbors (kNNs) and artificial neural network. The following subsection give a brief review of such classifiers. 

### 4.1. Support Vector Machine

 The SVM is a largely used classification technique introduced by Vapanick [35]. It learns how to discriminate between positive and negativ(in our case mass and non mass), by finding a hyperplane as a decision surface separating the classes. The hyperplane is defined by support vectors. The SVM uses an optimization method identifying the support vectors *s*_*i*_, the weights *a*_*i*_, and the bias *b* which are used for the classification of the vectors *x* according to the following equation:

(15) C(x)=∑iaiφ(si,x)+b,

where *φ* corresponds to a kernel function. *φ* refers to a dot product in the case of a linear kernel. Then, if *c* ≥ 0, *x* is classified as a member belonging to the first class. Otherwise, it is classified as a member belonging to the second class. 

### 4.2. k-Nearest Neighbors

 kNN classifier is a well-known method in a large number of applications. Since kNN is memory based, no models need to be trained. For a given instance *x*, the kNN first finds the *k* closest training points with respect to a particular distance metric. Then, it uses its labels in order to classify the instance *x* by majority vote [36]. In this study, we use the Euclidian distance to determine the nearest neighbors of the query element, and *k* is used as a training parameter. For each element *x*, the output score corresponds to the ratio of the winning class elements among the total number of neighborhood in the corresponding dataset. 

### 4.3. Artificial Neural Network

 ANN has been widely used in many applications where the expert knowledge is not clearly defined [37]. The idea of the ANN has been inspired from the biological nervous system and has been successfully applied in medical imaging. This technique is based on the adjustment of weights between the neurons for any input-output function approximation. Therefore ANN, has been widely used in digital mammography to mimic this computational power and the perception capabilities of human brain.

Two basic types of ANN, the multilayer perceptron (MLP) as well as the radial basis function network (RBF), are frequently used in recent works.

On the one hand, multilayer perceptrons (MLPs) are feedforward ANN models typically trained with static backpropagation. The MLPs find their way into many applications which require static pattern classification. Their principal advantage is the ease of use and the approximation capability of any input/output map.

On the other hand, radial basis function (RBF) networks are nonlinear hybrid networks containing a single hidden layer of processing elements. This layer uses gaussian transfer functions and the sigmoidal functions used by MLPs [38]. This type of ANN is in generally used when the number of samples is so small (<100). So, the limitation of the RBF neural network is that it is very sensitive to the dimensionality and has more and more difficulties if the number of units is large.

Based on this assumption, and knowing that the GLLD feature size is of 1352, we intend to investigate the MLP to exploit the results using the ANN [39]. Details of the used MLP network parameter are presented in Table 3. Let us consider *x* = (*x*1, *x*2,…, *xd*)^*T*^ the input vector, *ω* = (*ω*1, *ω*2,…, *ωd*)^*T*^, the weight vector, and *g*(*x*) = (1 + *e* ^−*x*^)^−1^ the activation function which corresponds to a sigmoid function, and the network output is thus defined as follows:

(16) y=g(ωTxb)=g(∑i=1dωixi−b).

**Figure 8:**
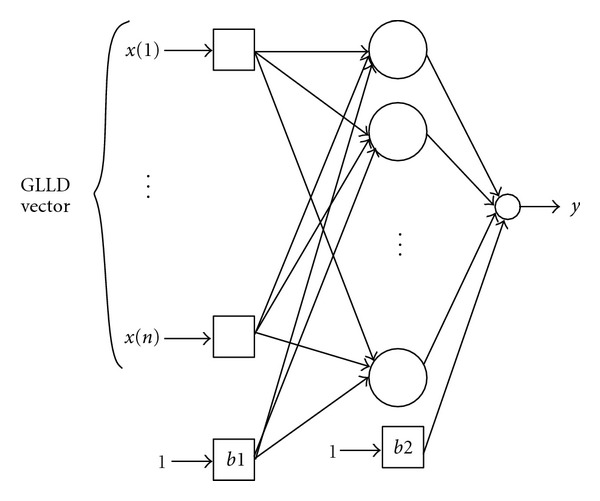
MLP classifier architecture.

For each ROI sample, GLLD features are computed and used in the classification step as inputs of the neural network.  Figure 8 illustrates the applied neural network. 

**Figure 9:**
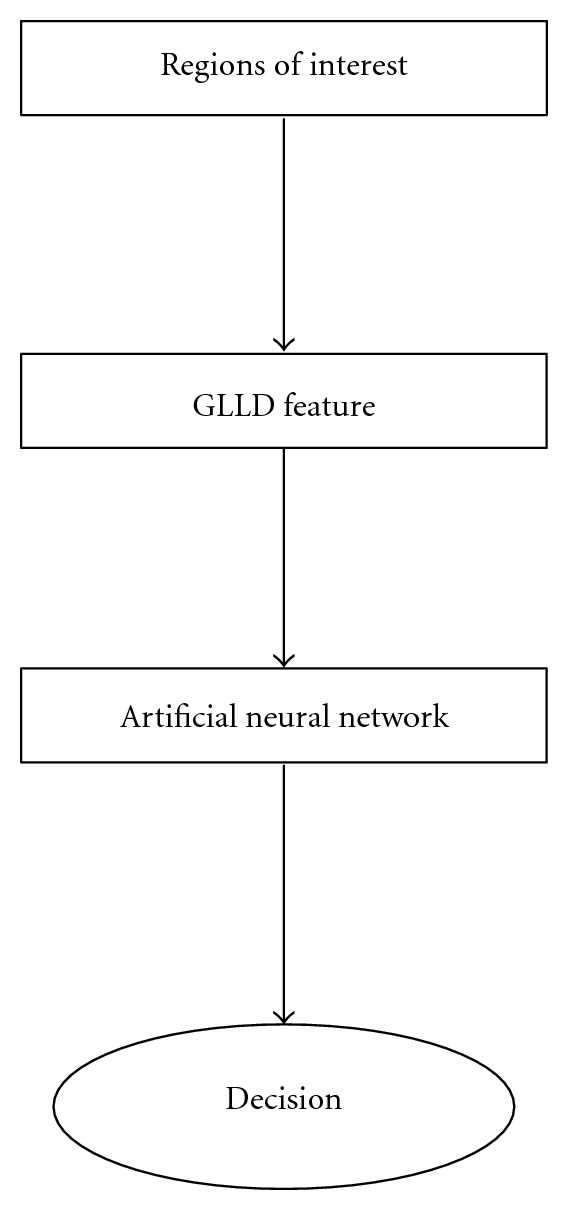
Implementation of the proposed method.

 The evaluation of the effectiveness of the training is based on the measure of the network relative error as follows:

(17) E=∑i=1n(y−T)number  of  applied  samples,

where *y* is the ROI corresponding to masses or nonmasses resulting from ANN and *T* corresponds to the target. The use of artificial network may lead to low error rates. After the training step, generalization errors may be evaluated for various features and network conditions.  Figure 9 maps the different steps of the proposed method. 

In the following section, the obtained results for ANN, SVM, and kNN classifiers will be illustrated for comparison purposes. 

## 5. Experimental Results

This section is composed of the following parts. First of all, the database used in the evaluation is presented. Afterwards, we illustrate the results for different rotation invariant rows under setting. We then do an investigation of the feature relevance, by using each of the proposed features (SGLLD, MGLLD, CGLLD) separately as input vector to the classifier. In further step, we made the classifier input a concatenated vector made up with different feature vectors. The obtained feature vector allow as to compare different methods of classification. Then, we made our experiments for different ROI image sizes. Finally, a comparative study of our proposal to those in the state-of-the-art will be done for a fair evaluation. 

### 5.1. Mammogram Dataset

Our approach has been evaluated based on publicly available database taken from the DDSM database [40].

DDSM contains 2620 individuals, available in 43 volumes. A volume corresponds to the collection of different cases. A case is the collection of all information to the mammography exam of one patient. Each case in the DDSM database contains two images, of each breast, that is, in each case the mammograms include a craniocaudal and mediolateral oblique view (CC and MLO, resp.). The DDSM database provides the metadata (date of study, breast density, assessment categories, etc.) of each abnormality using the breast imaging reporting and data System (BI-RADS) lexicon, it provides, also, the corresponding chain codes of the suspicious regions. With these chain codes, the outlines of the abnormalities may be identified. The DDSM provide delineations of mass regions. However, precision of such delineations is not adequate for validation in our approach, since it was done on downscaled images of DDSM database (by factor of 8) [41], see Section 2. Therefore, we wore based in the extraction of ROI's on manual segmentation entertained by two expert radiologists of more than 30 years of clinical experience from the Farabi imaging. We should also notice that all the considered masses in DDSM are biopsy proven ones.

### 5.2. Influence of Rotation Invariant Rows under Settings

The study was based on 1000 ROI extracted from mammograms from DDSM database. These ROIs were randomly selected and separated into two sets: 500 samples for training and 500 samples for tests. In the training set as well as in the setting set, we used 250 samples corresponding to masses and 250 samples corresponding to nonmasses. The evaluation of our mass detection algorithm is performed by applying a leave-one-out methodology, where the input ROI is classified by using the appropriate classification method and the procedure is reapplied for all the remaining ROIs used as input.

 From the results presented in Table 4, we can conclude that with (*P*, *R*) = (24,3), the area under curve for the GLLD is increased from 0.93 to 0.95. Rows under settings choice affect very slightly *A*_*z*_ performance. In the next experiments, GLLD_24,3_^riu2^ will be used. 

### 5.3. Investigation of the Method of Classification

 From the comparative study, as shown in Table 5, we note that the ANN provides the best results. This can be attributed to its higher performance as function approximator. 

### 5.4. Investigation of the Relevance of the Features

**Figure 10:**
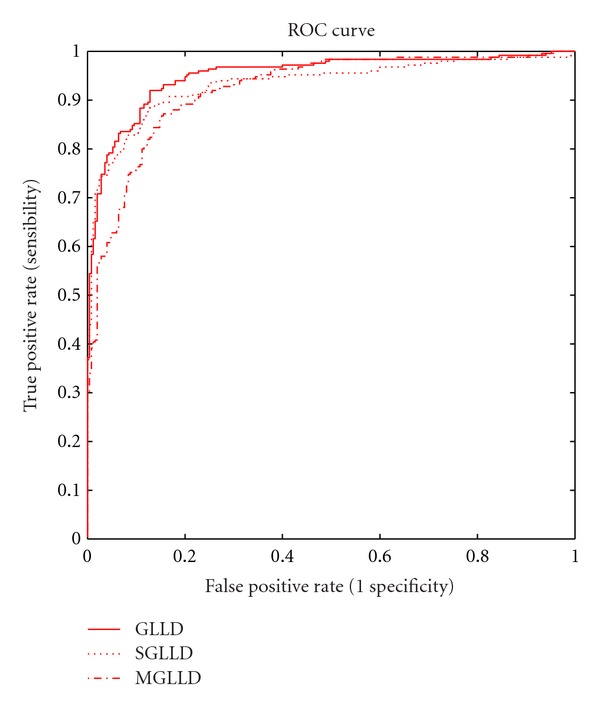
ROC curve corresponding to a subset of 1000 ROIs images from the DDSM database.

As illustrated in Figure 10 and Table 6 the CAD system achieves better performance (*A*_*z*_ = 0.93) when using the sign component than the modulus component. However, their fusion may provide much better results in texture classification than using either sign or modulus (*A*_*z*_ = 0.95). The AUC of the GLLD_24,3_^riu2^ after the fusion of the three operators SGLLD_24,3_^riu2^, MGLLD_24,3_^riu2^, and CGLLD_24,3_^riu2^ using the ANN as classifier is about (*A*_*z*_ = 0.95) for the experimental set. 

 As can be noted from Table 6, the GLLD_24,3_^riu2^ feature provides useful information about local gray level which is the most significant one for mass detection in mammographic images.

### 5.5. Results Varying the ROI Image Sizes

 Based on the size of the lesion, we use six different group of ROI images, which is an important aspect for the correct classification of the masses. These classes correspond to the following specified mass sizes intervals [9]: size 1: <10 mm^2^, size 2: (10–60) mm^2^, size 3: (60–120) mm^2^, size 4 : (120–190) mm^2^, size 5: (190,270) mm^2^, and size 6: >270 mm^2^. However, the used numbers of masses in each class size were, respectively, 28, 32, 37, 57, 69, and 33 masses.  Table 7 illustrates the *A*_*z*_ values for each class of ROI image sizes and the obtained mean *A*_*z*_ values. We include in this table a quantitative comparison with the work of Oliver et al. [8, 9] where the same sizes are considered. Oliver et al. in [8, 9] have used our database of ROI ratio (1/3), the same specified mass size intervals, and the same number of masses in each class size. 

 The results presented in Table 7 have shown that the GLLD_24,3_^riu2^ features are effective for mass detection at different ROI image sizes, and the latter is an important aspect for correct classification of the masses. Our method proves its performance in the most difficult case, which correspond to the smaller masses. Note that for this proposed ratio, better results are obtained for all the size intervals. 

### 5.6. Comparison with Some Consequent Approaches on Mass Detection in the State-of-the-Art

Table 8 shows the different *A*_*z*_ performance values for different approaches in the state-of-the-art presented in Section 2. Such which were represented comparison demonstrates the effectiveness of the proposed GLLD operator in mass detection. For instance, [10, 12, 13], which used ratio (1/1) the same as in our cases, obtained *A*_*z*_ values 0.83, 0.90, and 0.81, respectively, as our obtained *A*_*z*_ = 0.95. 

## 6. Conclusion

 CAD systems have been used and gained greater utility in recent years, as a second virtual reader for the medical images, contributing to increase an early detecting of breast cancer. This work presents a new method for mammographic mass detection based on textural features. Our proposal combines gray level as well as local differences. The combined descriptors are, respectively, SGLLD_24,3_^riu2^, MGLLD_24,3_^riu2^, and CGLLD_24,3_^riu2^ providing a final texture feature descriptor named GLLD, which will be used to classify the ROIs to masses and mass free. The ANN classifier gives better performances in term of classification owing to its higher function approximation. Different image sizes were considered for better improving detection rates. Finally, a comparative study with previous works was done for fairer evaluation. Such comparison illustrates that our proposed method leads to the best performance *A*_*z*_ = 0.95. The specialists who I have collaborated with found that the proposed CAD improved the sensitivity of mammography screening. In fact, CAD system is useful in situations where there is a high interobserver variability, lack of trained observers, or impossibility to perform the double reading with two or more radiologists as stated in the BIRADS categories. Future work will be focused in the classification of masses into the four Breast Imaging-Reporting and Data System (BI-RADS) categories.

## Figures and Tables

**Table 1:** Previously developed approaches on mass detection based on feature extraction and on learning. In this table, we specify for each approach the feature extraction technique, the classifier, the ratio which indicates the number of real masses/number of normal ROIs, and the obtained results. In the feature extraction methods, ICA, PCA, and 2DPCA correspond, respectively, to independent component analysis, principal component analysis and two-dimensional PCA. In the classification stage, ANN, NN, and SVM correspond, respectively, to the artificial neural network, nearest neighbors, and support vector machines. Generally, the evaluation of the works is given in terms of *A*_*z*_ where *A*_*z*_ represents the area under the ROC curve, except for both works of Christoyianni et al. and Leonardo et al. giving the correct classification true positive and true negative in percentage.

Classifier based											
Author	Year	Texture	Morphology	Shape	Gray level	ICA	PCA	2DPCA	Classifier	Ratios	Results
Qian et al. [6]	2001	√		√					ANN	200/600	*A*_*z*_ = 0.86
Christoyianni et al. [7]	2002	√			√		√		ANN	119/119	88.23%
Oliver et al. [8]	2006						√		C4.5 + NN	196/392	*A*_*z*_ = 0.83
Oliver et al. [9]	2007							√	NN	256/1536	*A*_*z*_ = 0.86
Varela et al. [10]	2007		√		√				ANN	60/60	*A*_*z*_ = 0.90
Leonardo et al. [11]	2009	√		√					SVM	250/1177	92.63%

**Table 2:** Previously developed approaches on mass detection based on template matching. In this table, the ratio which indicates the number of real masses/number of normal ROIs.

Template-matching based							
Author	Year	Gray level	Shape	Entropy	Similarity	Ratios	Results
Chang et al. [12]	2001		√	√	Likelihood function	300/300	*A*_*z*_ = 0.83
Tourassi et al. [13]	2007	√		√	Mutual function	901/919	*A*_*z*_ = 0.81

**Table 3:** Details of MLP network parameter.

Number	Functions used for MLP	Used parameters
4	Activation	Sigmoid function
5	Hidden Layer	1
	Number of hidden units	20
6	Input neurons	1352
7	Output neuron	1
8	Maximum mean square error	0.001
9	Number of iterations	2000

**Table 4:** Classification rates when using different number of rotation invariant rows under settings of (*P*, *R*) = (8,1), (*P*, *R*) = (16,2), and (*P*, *R*) = (24,3).

*P*, *R*	8, 1	16, 2	24, 3
*A*_*z*_	0.93	0.94	0.95

**Table 5:** *A*_*z*_ comparison of the different methods of classification (SVM, kNN, ANN) when utilizing GLLD as a feature extraction technique.

*A*_*z*_			
	kNN	SVM	ANN
GLLD_24,3_^riu2^	0.89	0.9	0.95

**Table 6:** *A*_*z*_ for different existing local pattern-based features and the GLLD proposed one.

	LBP + ANN	SGLLD_24,3_^riu2^ + ANN	MGLLD_24,3_^riu2^ + ANN	GLLD_24,3_^riu2^ + ANN
*A*_*z*_	0.89	0.93	0.92	0.96

**Table 7:** Obtained *A*_*z*_ values (ratio 1/3) of the classification of masses according to the ROI image sizes. The final column illustrates the mean *A*_*z*_ value. Size 1 to size 6 correspond to the different ROIs image sizes, from smaller to bigger one.

*A*_*z*_							
Method	Size 1	Size 2	Size 3	Size 4	Size 5	Size 6	Mean
Oliver et al. [8]	0.53	0.7	0.7	0.68	0.72	0.83	0.7
Oliver at al. [9]	0.81	0.83	0.87	0.84	0.89	0.93	0.86
GLLD_24,3_^riu2^ + ANN	0.98	0.99	0.97	0.92	0.9	0.93	0.94

**Table 8:** Presented *A*_*z*_ values for different methods in the state-of-the-art aiming at mass detection and that of the proposed one.

Method	Number of used ROIs	Ratio	*A*_*z*_
Qian et al. [6]	800	1/3	0.86
Chang et al. [12]	600	1/1	0.83
Varela et al. [10]	120	1/1	0.90
Oliver at al. [9]	1792	1/2	0.83
Tourassi et al. [13]	1820	1/1	0.81
GLLD_24,3_^riu2^ + ANN	1100	1/1	0.95
Human observers	1100	1/1	0.87
